# Response Letter: Are atypical lymphocytes a new predictive factor in the development of coronavirus disease 2019?

**DOI:** 10.1590/0037-8682-0309-2022

**Published:** 2022-09-19

**Authors:** Bruno Ramos Nascimento

**Affiliations:** 1 Universidade Federal de Minas Gerais, Hospital das Clínicas, Belo Horizonte, MG, Brasil.

**Dear Sir:**

First, thank you for your interest in our manuscript titled “Bedside Echocardiography to Predict Mortality of COVID-19 Patients Beyond Clinical Data: Data From the PROVAR-COVID Study^1^.” In this imaging study, we performed echocardiographic evaluation of patients with moderate to severe coronavirus disease 2019 (COVID-19) at the bedside at the earliest convenience, aiming to define the clinical, demographic, and echocardiographic predictors of mortality after adjusting for multiple variables, including comorbidities and risk factors. Left ventricular ejection fraction (LVEF) and tricuspid annulus systolic excursion (TAPSE) emerged as independent predictors of mortality in our analysis along with age, a classic and probably the most important prognostic factor of this disease, even after careful adjustments.

The role of imaging in defining the prognosis of patients with COVID-19 has been extensively explored since the inception of the pandemic in 2020. In preliminary efforts to define predictors of adverse outcomes, preexisting cardiovascular disease emerged as a key factor in predictive models^2^, as demonstrated in other viral diseases such as severe acute respiratory syndrome coronavirus 2 and Middle East respiratory syndrome coronavirus. This suggests that disease-induced myocardial injury by different mechanisms linked to viral infection^3^, stress due to systemic inflammation, and a prothrombotic state may potentially induce decompensation in patients with preexisting cardiac disease^4^. Thus, inflammation may play an important role as a trigger. As stated by the authors of this response letter, in addition to the preliminarily observed lymphopenia in patients with COVID-19, other morphological and functional changes in lymphocytes may be implicated in the dysregulation of the inflammatory cascade, which is strongly linked to outcomes.

Inflammation seems to play an important role in viral diseases with cardiac involvement, especially COVID-19, and magnetic resonance imaging (MRI) studies have highlighted this mechanism in part. A case series demonstrated that at least half of the patients (as high as 80%, depending on disease severity) admitted with COVID-19 had some abnormalities on MRI. Moreover, marked myocardial involvement was observed in nearly a third of the cases^5^ ^,^ ^6^, including gadolinium enhancement in 31%, as well as elevated global native T1, T2, and extracellular volume. Such observations were reaffirmed by a compilation of 34 preliminary reports (199 individuals), in which only 21% had normal MRI findings. Diffuse myocarditis was the most common diagnosis (40%), with a large proportion of T1 and T2 mapping abnormalities, myocardial edema, and subepicardial late gadolinium enhancement, which are markers of considerable tissue inflammation^7^. Such findings have been replicated by subsequent studies and highlight the complexity of cardiac involvement in patients with COVID-19, which possibly lies beyond simple functional impairment and may have an intrinsic connection with inflammatory patterns. Thus, revised markers of such processes are crucial, as proposed by the authors of this letter in reference to the lymphocyte panel.

Furthermore, MRI and computed tomography studies also suggest that the involvement of the left (LV) and right ventricles (RV) in patients with COVID-19 may be subtle, especially in the early phases of the disease and in patients with mild-to-severe presentations^1^ ^,^ ^8^. Some authors have demonstrated a more prevalent involvement of the RV and relative sparing of the LV, although the echo parameters utilized for this assumption varied from subjective functional evaluation to the free-wall strain and indirect measurements such as the TAPSE, as observed in our study. Overall, different variables linked to RV involvement are associated with elevated D-dimer and troponin levels, which are both markers of inflammation and a higher mortality risk with COVID-19. In addition, some studies have demonstrated that pulmonary hypertension is a stronger predictor of mortality than RV dysfunction, raising doubts about the actual pathological pathway that accounts for RV involvement^9^. Regardless, both direct ventricular involvement and pulmonary thrombosis and hyperreactivity are directly linked to endothelial dysfunction, abnormal vascular reactivity, and ultimately, exacerbated inflammation^1^ ^,^ ^8^. Thus, the interaction of such findings with variables linked to lymphocyte activity, morphology, and function may provide additional insights into the aforementioned mechanisms.

Finally, echocardiography does not seem to have optimal accuracy in the detection of ventricular involvement patterns in patients with COVID-19. The data suggest that subtle differences in LV and RV function in individuals with and without cardiac involvement as detected by cardiac MRI were not markedly captured by transthoracic echo when they were within or very close to the normal range, as observed in our LVEF and TAPSE measurements^5^. This can be even more challenging when studies are performed in suboptimal environments such as overwhelmed emergency departments or intensive care units, or with patients in hyperdynamic and hyperinflammatory states or taking inotropes. Thus, it is clear that the prognostic tools for COVID-19 need to be fine-tuned, with the incorporation of demographic, clinical, imaging, and, ultimately, hematological data. For the latter, novel markers such as those related to lymphocyte morphology and function may be promising tools, and more detailed longitudinal studies are warranted to achieve more definitive answers and develop optimal prognostic models.
